# Phytochemical Composition, Bioactive Compounds, and Antioxidant Properties of Different Parts of *Andrographis macrobotrys* Nees

**DOI:** 10.3390/life13051166

**Published:** 2023-05-11

**Authors:** Dayanand Dalawai, Hosakatte Niranjana Murthy, Yaser Hassan Dewir, Joseph Kadanthottu Sebastian, Anish Nag

**Affiliations:** 1 Department of Botany, Karnatak University, Dharwad 580003, India; 2 Department of Horticultural Science, Chungbuk National University, Cheongju 28644, Republic of Korea; 3 Plant Production Department, College of Food & Agriculture Sciences, King Saud University, Riyadh 11451, Saudi Arabia; 4 Department of Life Sciences, Christ University, Bangalore 560029, India

**Keywords:** *Andrographis*, antioxidants, GS-MS analysis, bioactive compounds

## Abstract

*Andrographis macrobotrys* Nees is an ethnomedicinal plant belonging to the family Acanthaceae, distributed in the moist deciduous and semi-evergreen forests of the southern Western Ghats of India. The objective of this research was to determine the phytochemical composition and bioactive chemical components using gas chromatography and mass spectrometry (GC-MS) and to check the antioxidant potential of the plant part extracts. *A. macrobotrys* roots, stems, and leaves were obtained from the species’ natural habitat in the Western Ghats, India. The bioactive compounds were extracted using a Soxhlet extractor at 55–60 °C for 8 h in methanol. Identification analysis of *A. macrobotrys* bioactive compound was performed using GC-MS. Quantitative estimation of phytochemicals was carried out, and the antioxidant capacity of the plant extracts was determined by 2,2′-diphenyl-1-picrylhydrazyl radical scavenging (DPPH) and ferric reducing assays (FRAP). *A. macrobotrys* has a higher concentration of phenolics in its stem extract than in its root or leaf extracts (124.28 mg and 73.01 mg, respectively), according to spectrophotometric measurements. GC-MS analysis revealed the presence of phytochemicals such as azulene, 2,4-di-tert-butylphenol, benzoic acid, 4-ethoxy-ethyl ester, eicosane, 3-heptadecanol, isopropyl myristate, hexadecanoic acid methyl ester, hexadecanoic acid, 1-butyl-cyclohexanol, 9,12-octadecadienoic acid, alpha-monostearin, and 5-hydroxy-7,8-dimethoxyflavone belonging to various classes of flavonoids, terpenoids, phenolics, fatty acids, and aromatic compounds. Significant bioactive phytochemicals include 2,4-di-tert-butylphenol, 2-methoxy-4-vinylphenol, 5-hydroxy-7,8-dimethoxyflavone, azulene, salvigenin, squalene, and tetrapentacontane. In addition, the antioxidant capability of each of the three extracts was assessed. The stem extract demonstrated impressive DPPH scavenging and ferric reduction activities, with EC_50_ values of 79 mg/mL and 0.537 ± 0.02 OD at 0.2 mg/mL, respectively. The results demonstrated the importance of *A. macrobotrys* as a source of medicine and antioxidants.

## 1. Introduction

Many secondary metabolites that plants produce have developed over time as protection against pathogens and herbivores. The use of secondary metabolites in pharmaceuticals, nutraceuticals, herbal cosmetics, and food supplements is widespread. Due to their advantages over synthetic pharmaceuticals, such as fewer side effects, better compatibility with human physiology, and lower prices, the demand for plant-based chemical compounds is rising globally [1]. The concept of linking a plant’s phytochemicals to its pharmacological activity is becoming increasingly popular. Hence, after identifying plants that are significant from an ethnopharmacological perspective, chemical compounds from those plants are extracted, isolated, and characterized employing highly sophisticated chromatography techniques, including gas chromatography and mass spectroscopy (GC-MS).

Acanthaceae is a family of plants that includes the genus *Andrographis* Wall. ex. Nees, which is composed of therapeutic plants. *Andrographis* is represented by 48 species that are annual and perennial herbaceous plants distributed in tropical regions of Asia. The majority of *Andrographis* species are endemic to India, and some of them are lesser known but significant medicinal plants utilized by native and tribal people in the Deccan Plateau and Western Ghats, particularly *A. paniculata*, often known as “King of Bitter,” which has many uses in modern medicine. It has a wide range of pharmacological actions, including anticancer, antidiabetic, hepatoprotective, anti-inflammatory, antiviral, antioxidative, and antibacterial activities [2]. Diterpenoids, including andrographolide, neoandrographolide, deoxyandrographolide, and 14-deoxy-11,12-didehydroandrographolide, as well as flavonoids, are thought to be responsible for its pharmacological effects [3,4]. Since ancient times, local communities and practitioners of traditional medicine have used a number of *Andrographis* species known for their ethnomedicinal properties to treat a variety of conditions, including wounds, fever, snake bites, constipation, jaundice, diabetes, skin diseases, and a number of other disorders [3,5]. It follows that members of the *Andrographis* genus have a great deal of potential for medicinal uses, and there is a lot of room for isolating and characterizing phytochemicals from lesser known species for use in medicine.

Due to its extensive therapeutic benefits, there is a significant demand for “*Andrographis*” raw material on a global scale. To accommodate this demand, *A. paniculata* has been cultivated in Asian nations. The overexploitation of *A. paniculata* natural populations, however, is one of the main causes of the species’ natural distribution becoming depleted. *A. macrobotrys* was identified by Dalawai et al. [4] as a potential substitute for the biosynthesis of neoandrographolide, a significant diterpenoid molecule. The neoandrographolide level in *A. macrobotrys* was 102.03 mg/g DW, while it was 11.72 mg/g DW in *A. paniculata* [4]. This suggests that *A. macrobotrys* has the potential to be the most effective source of neoandrorapholide and that it may also lighten the load on *A. paniculata*. In addition, only a few species, including *A. paniculata, A. echioides*, and *A. producta*, have been successfully identified as phytochemicals and their compositions using the GC-MS technique [5,6]. Other species, such as *A. macrobotrys*, have not yet had their phytochemical constituents thoroughly investigated.

In order to fully appreciate *A. macrobotrys* potential as a medicine, it is important to investigate and identify its chemical makeup. After this is accomplished, researchers can shift their focus to species conservation and sustainable use. Hence, using GC-MS techniques, the current study was aimed at investigating specific chemical components of the root, stem, and leaf of *A. macrobotrys*. Furthermore, in vitro techniques have been used to study the antioxidative qualities of methanolic extracts of roots, stems, and leaves.

## 2. Materials and Methods

### 2.1. Plant Materials

*A. macrobotrys* roots stems, and leaves were obtained from the species’ natural habitat near Hebri, Udupi, Karnataka, India (13.41789928, 74.94768698). An identification of the plant specimen was made using the Flora of the Presidency of Madras [7] and a voucher specimen (DSD-03) was deposited in the Shivaji University Herbarium in Kolhapur, India.

### 2.2. Chemicals and Reagents

Analytical-grade chemicals were employed in all of the tests. Methanol, sodium carbonate, sodium nitrate, ferric chloride, aluminium chloride, hydrochloric acid, 2,4,6-tripyridyl-s-triazine, 2,2-diphenyl-1-picrylhydrazyl (DPPH), butylated hydroxyl anisole, ascorbic acid, and Folin-Coicalteu (FC) reagents were procured from Sigma Aldrich Chemical Co. (St. Louis, MO, USA).

### 2.3. Methanolic Extract Preparation

The plant’s root, stem, and leaves were shade-dried before being separately blended to a fine powder. They were left for 24 h in a hot air oven to dry at 35 °C. Each part’s powder (100 g) was extracted with 100 mL of methanol using a Soxhlet extractor at 55–60 °C for 8 h. Each part’s extract was dried in a rotary evaporator under reduced pressure at 40 °C to remove any excess methanol before being used in further phytochemical and antioxidant studies.

### 2.4. Determination of Phytochemical Composition

In accordance with Folin and Ciocalteau’s [8] description of the spectrophotometric analysis, the amount of total phenolics in the methanolic extracts of the root, stem, and leaf were measured. The Folin–Ciocalteau (FC) reagent (1 mL) and 0.5 mL (1 g/mL) of the methanolic extracts were added to test tubes containing 2.5 mL of deionized distilled water. The mixture was given six minutes to stand before the addition of 0.5 mL 20% sodium carbonate solution. After 30 min of incubation at room temperature, the produced color’s absorbance was measured at 760 nm on a UV–visible spectrophotometer (UV-1601, Shimadzu, Kyoto, Japan). By comparing the results to the gallic acid standard curve, the amount of total phenolic content was determined and reported as mg gallic acid equivalent (GAE) per g dry sample.

Spectrophotometric analysis was used to assess the flavonoid content in the extracts of the root, stem, and leaf [9]. Each sample’s 0.5 mL of methanolic extract was combined with 2.5 mL of distilled water and 0.15 mL of sodium nitrite (5%) solution, and permitted to sit for 6 min. The reaction was then continued for another 5 min with the addition of 0.3 mL of 10% aluminium chloride. After mixing 2 mL of 1M sodium hydroxide, a spectrophotometer (UV-1601, Shimadzu) was used to read the absorbance at 510 nm. The amount of flavonoid content was calculated using the standard calibration curve of quercetin and reported as mg of quercetin equivalent (QE) per g of dry samples.

The methanolic extracts were used in the determination of tannins present in the samples spectrophotometrically according to the method developed by Schanderi [10]. Each extract (0.5 mL) was mixed with 2.5 mL of distilled water and 0.25 mL of Folin–Denis reagent, followed by 0.5 mL of 30% sodium carbonate. Then, all the reagents were mixed well to complete the reaction and incubated for 30 min at room temperature. The absorbance of the developed color was measured at 700 nm using a spectrophotometer (UV-1601, Shimadzu). The known amount of tannic acid was used to draw the calibration curve, and the amount of tannin in samples was determined and expressed as mg of tannic acid equivalent (TAE) per gram of dry samples.

### 2.5. Gas Chromatography and Mass Spectroscopy (GC-MS) Analysis

The chemical compounds present in the methanolic extracts of the samples were separated using gas chromatography and mass spectrometry (Model: QP2010S; Shimadzu Corporation, Kyoto, Japan), an advanced analytical device outfitted with Rxi-5Sil MS capillary column (length 30 m 0.25 mm ID, 0.25 m film thickness). The carrier gas, with a constant flow rate of 1 mL/min, was helium (99.9995%). The column oven temperature was originally maintained at 60 °C before being raised to 260 °C by 5 °C/min and maintained for 5 min. The split injection was used to inject the 1 L diluted samples, with a 4 min solvent delay. The show lasted 30 min in total. Temperatures of 200 °C and 280 °C, respectively, were selected for the interface line and ion source [11]. By contrasting the retention durations of real compounds with the mass spectra from the NIST 11 and WILEY 8 mass spectral libraries, the separated components were determined. Kovats retention index was determined using the structural formulae of the compounds as previously described [12]. For quality control of the gas chromatography analysis, we estimated a resolution between two peak separations using the formula: [{1.18(Rt2 − Rt1)}/(W2 + W1)], while Rt2 and Rt1 are the retention times of peak 2 and 1, respectively; W is the peak width. In additio, we estimated the signal-to-noise ratio for each of the sample runs, based on corresponding blank runs.

### 2.6. In Vitro Antioxidant Activity

The extracts’ capacity to scavenge DPPH radicals was assessed [13]. To make the stock solution, 24 mg of DPPH was dissolved in 100 mL of methanol. The working solution was created by combining methanol with DPPH stock solution to achieve an absorbance of around 0.99 ± 0.02 at 515 nm using a spectrophotometer (UV-1601, Shimadzu). A sample with a range of quantities (0.2–1.0 mg/mL) was combined with aliquots (3 mL) of this working solution (DPPH). After thoroughly shaking, the reaction mixture was allowed to sit at room temperature for 30 min in the dark. At 515 nm, the absorbance was then measured. The control was prepared concurrently without any sample. As standards, butylated hydroxyl anisole (BHA) and ascorbic acid were utilized. The following equation was used to obtain the DPPH scavenging activity percentage. [(Control OD − Sample OD)/Control OD] × 100 equals the percentage of DPPH scavenging activity.

The Benzie and Strain method was employed while performing the FRAP assay [14]. Plant extracts were added to 3 mL of newly made FRAP reagent (300 mM acetate buffer at pH 3.6, 10 mM 2,4,6-tripyridyl-s-triazine (TPTZ) in 40 mM HCl, and 20 mM FeCl_3_ in the ratio 10:1:1) at varied concentrations (0.2–1.0 mg/mL) and then heated to 37 °C in a hot water bath for 10 min. Distilled water was used to adjust the final volume to 4 mL, and it was incubated for 10 min at room temperature in the dark. At 593 nm, optical density was observed. As standards, butylated hydroxyl anisole and ascorbic acid were utilized. More antioxidant power was shown by an increase in optical density (OD).

### 2.7. Statistical Analysis

All experiments were conducted in triplicate using three different lots. The mean, percentage, EC50 values, and standard deviation were calculated using the statistical software SPSS, Version 17.

## 3. Results

### 3.1. Phytochemical Composition

In the root, stem, and leaf of *A. macrobotrys*, phytochemicals, including total phenolics, flavonoids, and tannins, were quantified and expressed as gallic acid equivalent (GAE), quercetin equivalent (QE), and tannic acid equivalent (TAE), respectively. The total phenolics, flavonoids, and tannins of different plant parts are shown in Table 1. The concentration of phenolics is the highest among all the plant parts.

### 3.2. Gas Chromatography and Mass Spectrometry (GC-MS) Analysis

**Figure 1:**
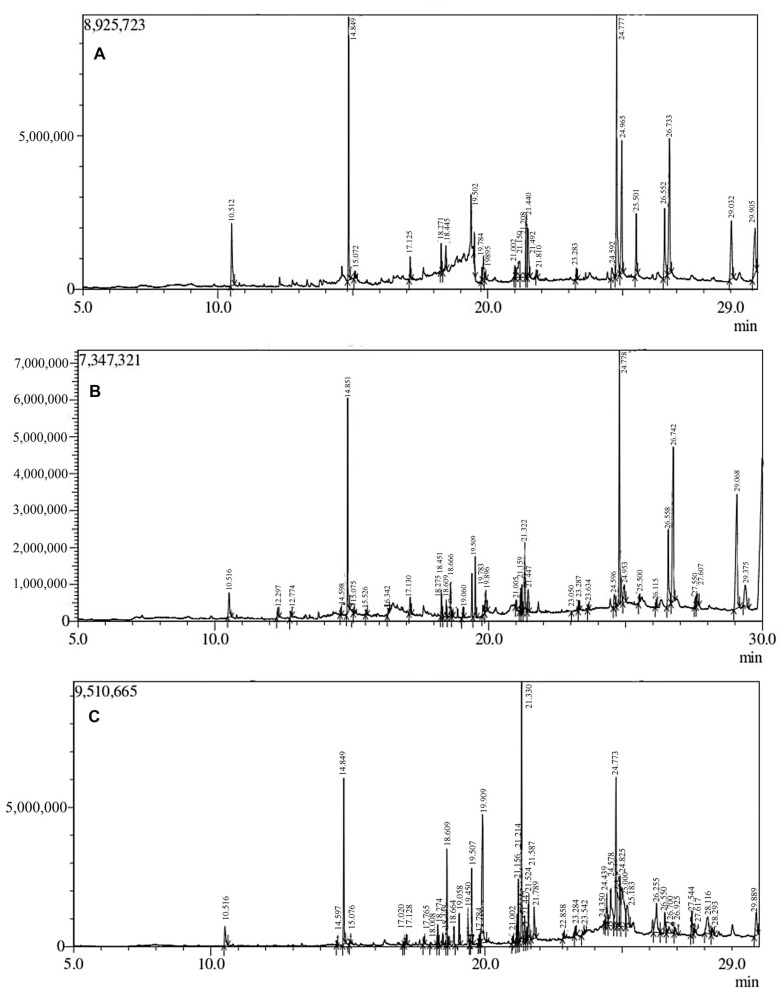
GCMS profile of the (**A**) root, (**B**) stem, and (**C**) leaves of *Andrographis macrobotrys*.

Extracts of the root, stem, and leaf of *A. macrobotrys* were subjected to gas chromatography and mass spectrometry (GC-MS) profiling to identify bioactive chemical compounds. Average resolution between two peaks was found to be ~1.0 for all GC runs reflecting 98% separation. We also noted a signal-to-noise ratio of around 5:1 for all the runs. These two parameters were well within the limit as stipulated by USP and ICH. The GC-MS analysis revealed the presence of various kinds of metabolites, including phenolics, sesquiterpenoids, isoprenoids, fatty acids, benzofurans, and aromatic compounds. The total number of compounds found in all the extracts was 104, including 24 from the root, 36 from the stem, and 44 from the leaf. The compounds from the root (Figure 1A), stem (Figure 1B), and leaf (Figure 1C) are presented in Table 2, Table 3 and Table 4, respectively. Additionally, a number of the phytochemicals in the extracts have been reported to be biologically active substances. Table 5 lists key bioactive substances that are present in the roots, stem, and leaves of *A. macrobotrys* and have been proven to exhibit biological activity.

### 3.3. In Vitro Antioxidant Potential

**Figure 2:**
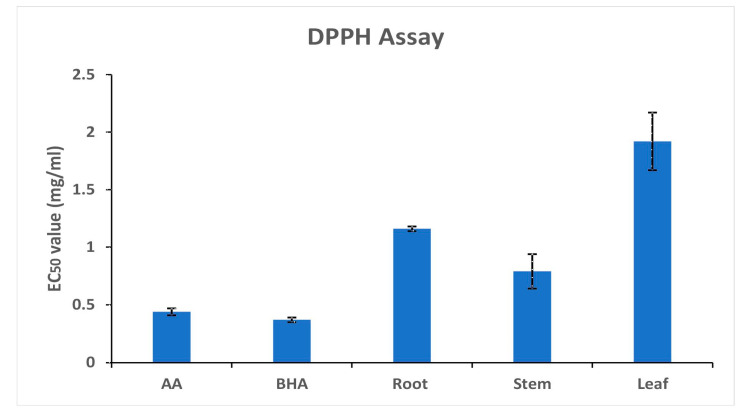
DPPH analysis of the root, stem, and leaves of *Andrographis macrobotrys*.

The dark purple color scheme of the DPPH free radicals is brought on by their single electron pair. Samples containing antioxidants decolorize the DPPH solution by scavenging the electrons. Thus, the reduction in the purple color of the DPPH solution is directly linked to the antioxidant capacities of the methanolic extracts of the samples as they scavenge the pair of electrons. The DPPH free radical scavenging activities of the extracts are expressed as their mg/mL EC_50_ value. The lower value of EC_50_ indicates higher radical scavenging activity. Ascorbic acid and BHA had EC_50_ values of 0.44 ± 0.03 mg/mL and 0.37 ± 0.02 mg/mL, respectively, whereas the root, stem, and leaf methanolic extracts had DPPH radical scavenging activities of 1.16 ± 0.2 mg/mL, 0.79 ± 0.15 mg/mL, and 1.92 ± 0.25 mg/mL, respectively (Figure 2).

**Figure 3:**
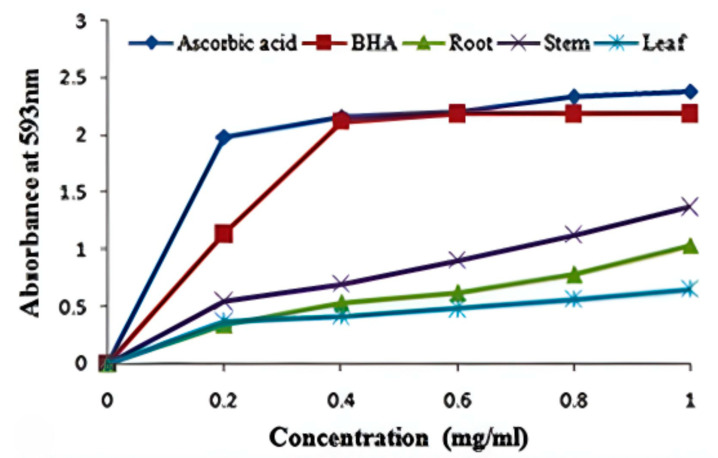
FRAP analysis of the root, stem, and leaves of *Andrographis macrobotrys*.

The colorless ferric-tripyridyltriazine (Fe3+-TPTZ) complex reduced to ferrous-tripyridyltriazine (Fe2+-TPTZ), an intense, blue-colored complex, by the activity of antioxidants present in the sample, due to the dose–response relationship. The sample containing higher levels of antioxidants may result in more intense color development. The methanolic extracts of the stem demonstrated the highest antioxidant activity, with ODs of 0.537 ± 0.02 and 1.367 ± 0.03, at concentrations of 0.2 and 1.0 mg/mL, respectively. The antioxidant capacity of various samples was in the following order: leaf, root, and stem (Figure 3). The activity of the extracts is comparable to that of ascorbic acid and BHA. The leaves and stem contributed to the highest phenolic and flavonoid content, which can be correlated with antioxidant activities.

## 4. Discussion

*A. paniculata*, the most popular species in the genus, is well known for its exceptional medicinal qualities [2,37]. It is therefore highly sought after in the pharmaceutical and herbal industries. Asian countries have used plant medications as an alternative to Western medicine. The plant is also well known for its incredible ability to combat viral infections. Because of this, demand for the raw materials and goods produced by *A. paniculata* increased dramatically during the COVID-19 outbreak [38]. The quest for alternatives with potential on par with *A. paniculata* has accelerated [39,40] in an effort to minimize the strain. The current phytochemical investigation was carried out on the closely similar species *A. macrobotrys* in order to confirm that, and it identified a variety of phytochemicals.

The investigation’s findings unambiguously show that *A. macrobotrys*’s stem extract (124.28 mg GAE/g) contains a higher amount of phenolics than the plant’s root or leaf extracts (73.01 mg GAE/g, respectively). Similar findings also applied to *A. paniculata*, where stem extracts revealed a higher phenolic content than leaves [41]. The amount of flavonoids in leaf extract was six times greater than that in root extract (9.06 mg QE/g), or 57.04 mg QE/gm. The best source of tannins was the stem (86.39 mg TAE/g), followed by the root (59.49 mg TAE/g) and leaf extract (57.33 mg TAE/g), which had roughly identical amounts. Our estimation shows that the stem is the best source of phytochemicals that are soluble in methanol when compared to the leaf and root. The root, stem, and leaf of *A. producta* all had varying amounts of phenolic, flavonoid, and tannin content in the methanolic extract [6], with the stem having the greatest levels (163.61 mg GAE/g) and TAE (84.52 mg/g) of phenolics and tannins, respectively. This further demonstrates the stem’s excellent status as a source of total phenolic chemicals. Furthermore, *A. macrobotrys* possessed the highest level of phenolic compounds (180.98 mg GAE/g), compared to the stems of *A. producta* [6], *A. paniculata* [42], and *A. echioides* [43].

*A. macrobotrys* methanolic extracts were chemically profiled by GC-MS, and the results showed that each extract included a variety of high- and low-molecular-weight metabolites in varied amounts. Twelve substances were included in all the extracts: azulene, 2,4-bis(1,1-dimethylethyl)-phenol, benzoic acid, 4-ethoxy-ethyl ester, eicosane, 3-heptadecanol, isopropyl myristate, hexadecanoic acid, methyl ester, hexadecanoic acid, 1-butyl-cyclohexanol; 9,12-octadecadienoic acid, methyl ester; alpha-monostearin; 5-hydroxy-7,8-dimethoxyflavone. The GC-MS profiling’s primary objective was to identify the biologically active compounds present in this crucial plant for medicine. The discovered bioactive substances exhibited similarities to the known *Andrographis* species [44]. *A macrobotrys* contains a lot of chemicals that are biologically active, as would be expected. In our previous studies [4], the plant was found to be a rich source of the bioactive compound Neoandrographolide (102.03 mg/g DW) through HPLC analysis, when compared to other *Andrographis* species.

The plant extracts showed significant antioxidant potential in comparison to the other *Andrographis* species [45]. The antioxidant and other biological activities of these plants were demonstrated by both crude and identified compounds. A dimer produced by the oxidation of 2,6-dimethoxy phenol (syringol) by laccase, which increased antioxidant activity for FRAP, TEAC, and DPPH by 119.32, 53.15, and 93.25%, respectively, in comparison to the substrate [19]. A flavoring ingredient known as 2-methyl-4-vinylphenol was shown to be a powerful anticancer agent that inhibited the migration of Panc-1 and SNU-213 pancreatic cancer cells as well as their viability by preventing the expression of their nuclear antigens [16]. A monoacylglycerol derivative known as 2-Monomyristin has shown antibacterial efficacy against an *Escherichia coli* bacterial stain [27]. Trimethoxy flavone and salvigenin at a dose of 25 M inhibited the oxidative stress-induced apoptosis in neuroblastoma SH-SY5Y cells by activating antioxidant factors in the neuroprotective assessment [30] (Table 1). According to Serino et al. [31], salvigenin exhibits biological activity, lowering lipid levels (−22.5% palmitic acid biosynthesis at 30 M) while increasing mitochondrial functionality (+15.4% at 30 M). With a MIC value of 125 g/mL, Aromadendrene, a terpenoid found in *Eucalyptus globulus*, was discovered to be particularly efficient against methicillin-resistant *Staphylococcus aureus* [35] (Table 1). According to Murata et al. [22], the carotenoid metabolite loliolide functions as a strong endogenous inducer and mediates the host’s defense reaction against three herbivores. In studies using 7,12-dimethylbenzanthracene to produce skin tumors, squalene (5%) was found to have an anticancer effect and to have reduced 26.67% of tumors in the test group [32] (Table 1). However, several chemical compounds such as 4,22-stigmastadiene-3-one, 3,4-dihydro-2(1h)-isoquinoline carboxamidine, 4,22-stigmastadiene-3-one, acetosyringone, alpha-monostearin, eicosane, geranyl linalool isomer, and tetrapentacontane belonging to various classes of phytochemicals need to be examined in detail for their biological activities.

Due to their protective properties, various plant species and their parts have been employed to cure chronic ailments. These medicinal herbs’ protective function is related to their chemical components, which also have antioxidant activities [46]. Polyphenols from plant sources, including phenolic acids and flavonoids, demonstrate effective antioxidant action [47]. With an EC_50_ value of 0.79 mg/mL, the methanolic extract from *A. macrobotrys* stem displayed the highest DPPH radical scavenging activity and is comparable to the EC_50_ values of the reference substances evaluated, such as ascorbic acid (0.44 mg/mL) and BHA (0.37 mg/mL) (Figure 2). This could be attributed to the higher phenolic content in the stem compared to other parts. As compared to ascorbic acid (3.22 mg/mL EC_50_), the stem of *A. producta* showed good DPPH radical scavenging action with a value of 3.58 mg/mL [6]. The stem of *A. macrobotrys* performed better than *A. producta* at scavenging DPPH radicals. Antioxidants from stems were shown to have comparable reducing activity to BHA (1.129 ± 0.02 OD at 0.2 mg/mL) and the highest Fe3+-TPTZ reducing potential (1.367 ± 0.03 OD at 1.0 mg/mL concentration). However, the decrease in Fe3+-TPTZ complex was substantially less active with root and leaf extracts (Figure 3). At 1.0 mg/mL concentration, *A. producta*’s ferric-reducing activity was determined to be 1.742 ± 0.02 OD (stem), 1.139 ± 0.03 OD (root), and 0.866 ± 0.016 OD (leaf) [6]. As of right now, the stem of *A. macrobotrys* (1.367 ± 0.03 OD) reduces ferric oxide more effectively than the root (1.139 ± 0.03 OD) and less effectively than the stem of *A. producta* (1.742 ± 0.02 OD). The root, stem, and leaf of *A. macrobotrys* exhibit the same antioxidant potential according to both DPPH and FRAP experiments. Due to its phenolic concentration and bioactive chemicals, such as 2,4-di-tert-butylphenol, syringe, squalene, and tetrapentacontane, the stem may have a high antioxidant potential. Since *A. macrobotrys* possesses valuable elements that account for antioxidant activity, it could be exploited in food and pharmaceutical preparations. The antioxidant potential of phytochemicals plays an essential role in bioactive food and pharmaceutical resources [48].

## 5. Conclusions

Various parts of *Andrographis macrobotrys* were analyzed quantitatively to determine the distribution and variety of phytochemicals. From the various plant components, large amounts of phenolics, flavonoids, and tannins were recovered. Due to the presence of phenolic chemicals, this study demonstrated that plant components have high antioxidative potential. According to GC-MS analysis, there were several key bioactive phytochemicals present, including 2,4-di-tert-butylphenol, 2-methoxy-4-vinylphenol, 5-hydroxy-7,8-dimethoxyflavone, azulene, salvigenin, squalene, and tetrapentacontane. Numerous other substances have been found for biological evaluation to understand their importance, including 4,22-stigmastadiene-3-one, 3,4-dihydro-2(1h)-isoquinoline carboxamidine, 4,22-stigmastadiene-3-one, acetosyringone, and alpha-monostearin, among others. Therefore, *A. macrobotrys* is a source of practical phytochemicals, as the present investigation showed. The plants can be utilized as an alternative to *A. paniculata*, preventing their overuse and extinction.

## Figures and Tables

**Table 1:** Phytochemical composition of *Andrographis macrobotrys* plant extracts.

Plant Part	Phenolics (mg GAE/g DW)	Flavonoids mg QE/g DW	Tannins (mg TAE/g DW)
Root	124.28 ± 0.24 ^b^	9.06 ± 0.79 ^c^	59.49 ± 0.22 ^b^
Stem	180.98 ± 0.76 ^a^	18.04 ± 0.08 ^b^	86.39 ± 0.06 ^a^
Leaf	73.01 ± 0.35 ^c^	57.04 ± 0.43 ^a^	57.33 ± 0.14 ^b^

Values are mean ± standard deviation (*n* = 3). Mean values followed by different superscript in a column are significantly different (*p* < 0.05) according to Duncan’s multiple range test.

**Table 2:** GC-MS profile of *Andrographis macrobotrys’* root.

Sl. No.	Compound Name	Retention Time	Concentration (%)
1	Azulene	10.512	4.71
2	2,4-Di-tert-butylphenol	14.849	11.46
3	4-Ethoxy-ethyl esterbenzoic acid	15.072	0.28
4	Eicosane	17.125	1.02
5	3-Heptadecanol	18.271	1.28
6	Isopropyl myristate	18.445	1.26
7	Hexadecanoic acid, methyl ester	19.502	0.93
8	2,3-Dimethyl-3-hexanol	19.784	0.57
9	Hexadecanoic acid	19.895	0.36
10	1-Butyl-cyclohexanol	21.002	0.59
11	Methyl octadeca-9,12-dienoate	21.150	2.73
12	6-Octadecenoic acid, methyl ester, (Z)-	21.208	10.02
13	Tetratriacontane	21.440	5.64
14	4,4′-Thiobis[2-(1,1-dimethylethyl)-5-methyl-phenol	21.492	3.79
15	2,6,10,14-Tetramethyl-hexadecane	21.810	0.34
16	Dotriacontane	23.283	0.55
17	2-Ethylbutyric acid, eicosyl ester	24.592	1.05
18	Hexadecanoic acid, 2-hydroxy-1-(hydroxymethyl) ethyl ester	24.777	15.47
19	Asaraldehyde	24.965	7.99
20	Acetosyringone	25.501	3.23
21	alpha-Monostearin	26.552	5.04
22	5-Hydroxy-7,8-dimethoxyflavone	26.733	10.65
23	5-Hydroxy-6,7,4′-trimethoxyflavone/salvigenin	29.032	5.55
24	3,5-Dihydroxy-6,7,8-trimethoxyflavone	29.905	5.50

**Table 3:** GC-MS profile of *Andrographis macrobotrys’* stem.

Sl. No.	Compound Name	Retention Time	Concentration (%)
1	Azulene	10.516	2.48
2	2-Methoxy-4-vinylphenol	12.297	0.66
3	2,6-Dimethoxy-phenol or syringol	12.774	0.45
4	Nonadecane	14.598	0.29
5	2,4-Di-tert-butylphenol	14.851	11.24
6	Ethyl 4-ethoxybenzoate	15.075	0.28
7	4-Methyl-2,5-dimethoxybenzaldehyde	15.526	0.26
8	N-Phenyl aniline	16.342	0.44
9	Eicosane	17.130	0.94
10	3-Heptadecanol	18.275	1.29
11	Isopropyl myristate	18.451	0.69
12	Neophytadiene	18.609	1.52
13	6,10,14-Trimethyl-2-pentadecanone	18.666	0.30
14	3,7,11,15-Tetramethyl-2-hexadecen-1-ol	19.060	0.55
15	Hexadecanoic acid, methyl ester	19.509	3.09
16	3-Ethyl-3-pentanol	19.783	1.03
17	Hexadecanoic acid	19.896	1.80
18	1-Butyl-cyclohexanol	21.005	0.47
19	9,12-Octadecadienoic acid, methyl ester	21.159	1.28
20	8,11,14-Docosatrienoic acid, methyl ester	21.216	1.52
21	Phytol	21.322	4.13
22	Octadecanoic acid, methyl ester	21.447	1.66
23	2-Mono-myristin	23.050	0.61
24	Tetrapentacontane	23.287	0.53
25	Octadecane	23.634	0.24
26	Ethyl 3-hydroxytridecanoate	24.596	1.33
27	Hexadecanoic acid, 2-hydroxy-1-(Hydroxymethyl) ethyl ester	24.778	16.03
28	Asaraldehyde	24.953	1.95
29	3-Acetyl biphenyl	25.500	0.42
30	N-{4-[2-(1,1-Dimethylethyl)-5-oxo-1,3-dioxolan-4-Yl]butyl}formamide	26.115	0.41
31	alpha-Monostearin	26.558	6.51
32	5-Hydroxy-7,8-dimethoxyflavone	26.742	15.35
33	Squalene	27.550	0.81
34	Stigmasta-5,22-dien-3-ol	27.607	1.39
35	5-Hydroxy-6,7,4′-trimethoxyflavone/Salvigenin	29.068	14.53
36	(3 beta,24S)-Stigmast-5-en-3-ol	29.375	3.53

**Table 4:** GC-MS profile of *Andrographis macrobotrys’* leaf.

Sl. No.	Compound Name	Retention Time	Concentration (%)
1	Azulene	10.516	1.62
2	Nonadecane	14.597	0.19
3	2,4-Di-tert-butylphenol	14.849	6.92
4	Ethyl 4-ethoxybenzoate	15.076	0.17
5	1-{2-[3-(2-Acetyloxiran-2-yl)-1,1-dimethylpropyl]cycloprop-2-enyl}ethenone	17.020	0.14
6	Eicosane	17.128	0.46
7	Tetradecanoic acid	17.765	0.36
8	Loliolide	18.008	0.24
9	3-Heptadecanol	18.274	0.86
10	Isopropyl myristate	18.448	0.46
11	Neophytadiene	18.609	3.91
12	1-Dodecanol, 3,7,11-trimethyl-	18.664	0.40
13	3,7,11,15-Tetramethyl-2-hexadecen-1-ol	19.058	1.18
14	Methyl palmitoleate	19.450	0.26
15	Hexadecanoic acid, methyl ester	19.507	3.16
16	3-Ethyl-3-pentanol	19.784	0.32
17	Phytane	19.817	0.28
18	Hexadecanoic acid	19.909	10.19
19	1-Butyl-cyclohexanol	21.002	0.36
20	9,12-Octadecadienoic acid, methyl ester	21.156	1.22
21	9,12,15-Octadecatrienoic acid, methyl ester	21.214	2.76
22	Phytol	21.330	12.21
23	Octadecanoic acid, methyl ester	21.447	1.40
24	9,12-Octadecadienoic acid	21.524	0.81
25	cis,cis,cis-7,10,13-Hexadecatrienal	21.587	2.65
26	Octadecanoic acid	21.789	1.83
27	3-Cyclopentylpropionic acid, 2-dimethylaminoethyl ester	22.858	0.26
28	Tetrapentacontane	23.284	0.44
29	Eicosanoic acid	23.542	0.18
30	3-Cyclopentylpropionic acid, 2-dimethylaminoethyl ester	24.350	0.17
31	3,4-Dihydro-2(1h)-isoquinolinecarboxamidine	24.439	2.07
32	2-Ethylbutyric acid, eicosyl ester	24.578	4.99
33	Hexadecanoic acid, 2-hydroxy-1-(hydroxymethyl) ethyl ester	24.773	8.99
34	3,7,11-Trimethyl-2,6,10-dodecatrien-1-ol,	24.825	3.93
35	Geranyl linalool isomer	25.000	7.44
36	2,6,10,14,18-Pentamethyl-2,6,10,14,18-icosapentaene	25.183	3.08
37	4,22-Stigmastadiene-3-one	26.255	3.79
38	alpha-Monostearin	26.550	1.29
39	5-Hydroxy-7,8-dimethoxyflavone	26.700	0.89
40	(3 beta)-Cholest-5-en-3-ol	26.925	0.57
41	Squalene	27.544	1.64
42	(3beta)-Stigmast-5-en-3-ol	28.116	2.76
43	Aromadendrene	28.293	0.58
44	3,5-Dihydroxy-6,7,8-trimethoxyflavone	29.889	2.33

**Table 5:** Important bioactive compounds detected by GCMS analysis in the root, stem, and leaves of *A. macrobotrys* having potential biological activities.

Bioactive Compounds	Root	Stem	Leaves	RT	KI	Biological Activity	References
Azulene	+	+	+	10.516	518.92	Anti-inflammatory	[15]
2-Methoxy-4-vinylphenol	−	+	−	12.297	905.88	Anticancer, anti-inflammatory, and antioxidant	[16,17,18]
2,6-Dimethoxy-phenol or Syringol	−	+	−	12.774	1000	Antioxidant	[19]
2,4-Di-tert-butylphenol	+	+	+	14.849	1372.27	Anticancer, anti-inflammatory, and antioxidant	[20]
Eicosane	+	+	+	17.128	1725.4	anti-inflammatory, analgesic, and antipyretic	[21]
Loliolide	−	−	+	18.008	1849.32	Herbivore resistance	[22]
Neophytadiene	−	+	+	18.609	1930.51	Anti-inflammatory	[23]
Hexadecanoic acid, methyl ester	+	+	+	19.507	2047.07	Anti-Inflammatory	[24]
Phytol	−	+	+	21.322	2267.1	Anti-inflammatory and immunomodulating	[25,26]
2-Mono-myristin	−	+	−	23.05	2459.83	Antimicrobial	[27]
Tetrapentacontane	−	+	−	23.287	2485.13	Antimicrobial, antioxidant	[28]
Asaraldehyde	+	−	−	24.965	2657.22	Anti-obesity	[29]
5-Hydroxy-7,8-dimethoxyflavone	+	+	+	26.733	2826.44	Neuroprotective and lipid lowering	[30,31]
Squalene	−	+	+	27.544	2900.36	Antioxidant, antitumor, and colon cancer	[32,33]
Stigmasta-5,22-dien-3-ol	−	+	−	27.607	2906.28	Antimicrobial	[34]
Aromadendrene	−	−	+	28.293	2966.72	Antibacterial activity	[35]
5-Hydroxy-6,7,4′-trimethoxyflavone/Salvigenin	+	−	−	29.032	3030.49	Neuroprotective and lipid lowering	[30,31]
3,5-Dihydroxy-6,7,8-trimethoxyflavone	+	−	−	29.905	3103.76	Antitumor	[36]

+ or − represents presence or absence of the bioactive compound; RT = Retention time, KI = Kovats retention Index.

## Data Availability

All data are presented in the article.
